# Exploring the Clinical Impact of RANK Pathway Inhibition in Advanced Breast Cancer: Insights From a Retrospective Study on CDK4/6 Inhibitors and Antiresorptive Therapy

**DOI:** 10.7759/cureus.63362

**Published:** 2024-06-28

**Authors:** Goncagul Akdag, Akif Dogan, Sedat Yildirim, Oguzcan Kinikoglu, Muhammed Edib Mokresh, Omar Alomari, Ezgi Turkoglu, Deniz Isik, Heves Sürmeli, Tugba Basoglu, Ozlem N Sever, Hatice Odabas, Mahmut E Yildirim, Nedim Turan

**Affiliations:** 1 Medical Oncology, Kartal Dr. Lütfi Kirdar City Hospital, Health Science University, Istanbul, TUR; 2 Medical School, Hamidiye International School of Medicine, University of Health Sciences, Istanbul, TUR

**Keywords:** metastatic hormone positive breast cancer, denosumab and cancer, cdk4/6 inhibitor, rank and rankl, breast cancer outcomes

## Abstract

Background and objective: Breast cancer (BC) remains a significant health concern, particularly in advanced stages where the prognosis is poor. The combination of endocrine therapy (ET) with cyclin-dependent kinase 4/6 inhibitors (CDK4/6i) has improved outcomes for advanced BC (aBC) patients. However, resistance to CDK4/6i remains a challenge, with no validated biomarkers to predict response. The receptor activator of the nuclear factor-kB (RANK) pathway has emerged as a key player in aBC, particularly in luminal BC. RANK overexpression has been associated with aggressive phenotypes and resistance to therapy. In view of these findings, we proceeded to investigate the potential involvement of the RANK pathway in luminal BC resistance to CDK4/6i. The objective was to evaluate the effectiveness of denosumab in increasing overall survival (OS) and progression-free survival (PFS).

Methods: In this retrospective analysis, 158 BC patients with bone metastases were included. Patients with human epidermal growth factor receptor-2 (HER2)-negative and hormone receptor-positive BC who received palbociclib or ribociclib in addition to antiresorptive medication were included. Patients received either denosumab or zoledronic acid (ZA) therapy. The primary endpoint was OS, with PFS as a secondary endpoint.

Results: Although the PFS and OS of denosumab were better than ZA in this study, it did not show a significant difference between the two drugs. Meanwhile, mOS was not achievable in patients in the denosumab group, while it was 34.1 months in patients in the ZA group. The hazard ratio (HR) showed a significant improvement for the denosumab group in patients under 60 of age (HR: 0.33, p<0.01), patients with a score of 1 HER2 overexpression (HR: 0.09, p=0.01), and patients with resistant endocrine (HR: 0.42, p=0.02) compared to ZA.

Conclusion: This study highlights the potential clinical relevance of the RANK pathway in BC treatment, and our findings suggest that denosumab may offer significant benefits in terms of PFS and OS for certain subgroups, particularly those with HER2 scores of 1, patients under 60, and those with endocrine-resistant BC. In conclusion, considering that RANK pathway status may be a predictive biomarker for CDK4/6i treatment and may cause treatment resistance, our results demonstrate the clinical relevance of the combination of CDK4/6i + ET with RANKL inhibition.

## Introduction

Breast cancer (BC) is a heterogeneous disease, with approximately 70% of cases belonging to the luminal subtype. This subtype is characterized by the expression of estrogen receptor (ER) and/or progesterone receptor (PR). Despite having a better prognosis in the early stages, these individuals have a terrible prognosis in advanced disease (four-year survival rate: 35.9) [1]. The combination of endocrine therapy (ET) with cyclin-dependent kinase 4/6 inhibitors (CDK4/6i) represents the current standard of care for the treatment of advanced ER+ human epidermal growth factor receptor-2 (HER2)-negative BC (aBC). This treatment approach has been demonstrated to significantly improve outcomes in this setting [2]. It is unfortunate that up to 20% of patients are unresponsive to therapy and that the majority develop clinical resistance within two years of starting treatment. Currently, there are no validated biomarkers to predict the response to CDK4/6i. Therefore, there is a clear unmet need for strategies to predict and overcome intrinsic resistance to CDK4/6i or to extend benefit by delaying acquired resistance [3].

Over the past decade, the receptor activator of the nuclear factor-kB (RANK) pathway has emerged as a significant mediator of both breast morphogenesis and carcinogenesis [4-6]. Nevertheless, the significance of the RANK pathway in the context of luminal BC has only recently been acknowledged. The findings indicate that RANK overexpression is associated with an aggressive luminal BC phenotype, characterized by a decreased proliferation rate and increased susceptibility to chemotherapy and ET [7]. Furthermore, it was demonstrated that ectopic RANK expression in non-transformed mammary epithelia results in a delayed onset of subsequent aggressive luminal-like tumors [8].

Bone metastases (BM) are a common site of tumor burden for many solid tumors. Osteoclast inhibitors, known as antiresorptive agents, bone-modifying agents, or bone-targeted agents, including bisphosphonates and denosumab, play a crucial role in significantly decreasing the occurrence of skeletal-related events (SREs) and postponing their onset in patients with BM from various cancer types [9]. In aBC, international practice guidelines have refrained from expressing a preference for a specific agent [10-12]. In consideration of the evidence that monthly denosumab offers a marginal enhancement in SRE reduction and analgesic effects and that denosumab can be administered subcutaneously rather than intravenously, it may be more advantageous to employ monthly zoledronic acid (ZA) than monthly denosumab [13]. However, the decision to use denosumab should be weighed against its significantly higher drug costs, along with evidence indicating the noninferiority of three-monthly ZA dosing compared to monthly dosing [14-16].

Despite their widespread use, questions regarding the improvement of overall survival (OS) have remained unanswered. The management of patients with metastatic bone disease necessitates an integrated and multidisciplinary approach, in conjunction with the use of antiresorptive agents. Antineoplastic treatment forms the cornerstone of management. In light of these findings, we proceeded to investigate the potential involvement of the RANK pathway in luminal BC resistance to CDK4/6i. Our objective was to evaluate the effectiveness of denosumab compared to ZA in increasing OS and progression-free survival (PFS).

## Materials and methods

Study design and criteria for patient inclusion

This retrospective analysis included patients with HER2-negative and hormone receptor-positive aBC and BM and patients receiving palbociclib or ribociclib in addition to antiresorptive medication in the oncology center of Kartal Dr. Lütfi Kırdar City Hospital between 2015 and 2024. Breast tumors that were both ER-positive and HER2-negative were defined as having more than 10% ER activity and negative HER2 test results (immunohistochemistry score 0 or 1+ or negative, and negative staining by dual-probe in situ hybridization). Patients over 18 with clinically confirmed primary BC and no concurrent malignancies were eligible for inclusion. Data on demographics, tumor histopathology, the presence of metastasis, and antiresorptive therapies were collected and assessed. Patient data were obtained retrospectively from patient records after obtaining written consent from the patients or their relatives.

Antineoplastic and antiresorptive therapy

Patients receiving antiresorptive therapy were administered either a 120 mg subcutaneous injection of denosumab or a 4 mg intravenous injection of ZA every four weeks. ZA dosage was adjusted based on creatinine clearance (for those with a baseline value of less than 60 mL/min), and treatment was interrupted if renal function deteriorated during the study (until serum creatinine returned to within 10% of baseline values). No dose adjustment was required for denosumab. Daily supplementation with calcium (≥500 mg) and vitamin D (≥400 IU) was recommended.

Palbociclib 125 mg (capsule form) and ribociclib 600 mg were administered once daily for three weeks, followed by a seven-day interval, which was repeated every 28 days (in conjunction with fulvestrant or continuous aromatase inhibitor treatment and a gonadotropin-releasing hormone agonist if pre- or perimenopausal female). Therapy was continued until disease progression or intolerable toxic effects. Palbociclib was reduced to 100 mg/day or 75 mg/day, while ribociclib was adjusted to 400 mg/day or 200 mg/day based on tolerability.

Statistical analysis

We conducted statistical analyses using R version 4.3.3 (The R Foundation, https://www.r-project.org/) along with several statistical packages, including "tidyverse," "meta," "survminer," "ggsurvfit," and "forestplotter." Descriptive statistics were utilized to present frequency distributions of data collected on antiresorptive therapy. Our primary endpoint was OS, defined as the time from CDK4/6i initiation to death from any cause. The secondary endpoint was PFS, defined as the period from the initiation of CDK4/6i therapy to the date of radiological progression or the most recent outpatient follow-up date. All data collected from 2010 to 2023 was included in the analysis. Our analysis presents the results of an early stage, with ongoing follow-up regarding OS. To estimate time-to-event endpoints, we employed the Kaplan-Meier method, with significance determined using the log-rank test. Additionally, we applied a Cox regression model to estimate hazard ratios (HR) for prognostic factors, both with and without adjustment, to identify potential treatment interactions. Subgroups were predefined based on age group, PR status, CDK4/6i treatment group, HER2 overexpression, CDK4/6i treatment line, visceral metastasis status, and endocrine-resistant status. Our significance level was set at ≤0.05, and we reported HR alongside their respective 95% confidence intervals (95% CI) for each antiresorptive therapy. Sample size analysis has been done in the early stages of this study. The results showed that we need group sample sizes of 48 and 96, respectively, to reliably (with a probability greater than 0.8) detect an effect size of δ≥0.5, assuming a two-sided criterion for detection that allows for a maximum type I error rate of α=0.05 (using R package version 0.3.1, Statistical Power and Sample Size Calculation Tools; The R Foundation, https://www.r-project.org/) [17].

Ethical approval

The study conducted with human participants followed ethical standards outlined by both the institutional and national research committees, as well as the principles established in the 1964 Helsinki Declaration and its subsequent amendments or equivalent ethical norms. Approval for the research was granted by the Institutional Review Board of Kartal Dr. Lütfi Kirdar City Hospital in Istanbul, Turkey, under approval number 2023/514/250/2 dated May 29, 2023.

## Results

In this study, a total of 158 patients were enrolled, with 57 (36.1%) receiving denosumab treatment and 101 (63.9%) receiving ZA. Considering the use of CDK4/6i, 84 (53.2%) of the patients were using ribociclib, while 74 (46.8%) were using palbociclib. Eighty patients (50.6%) were aged 60 or older. The histological types of aBC varied within the study, with invasive ductal carcinoma (45.6%) and infiltrating carcinoma (38.0%) being the most common types observed. ER status was assessed in 150 patients, with 100 (66.7%) showing expression greater than 90%. PR status was positive in 138 patients (87.3%). HER2 overexpression was evaluated in 148 patients, with 71 (48.0%) having a score of 0. Additional baseline characteristics are summarized in Table 1.

**Table 1:** Clinicopathological characteristics of the patients ECOG: Eastern Cooperative Oncology Group, ER: estrogen receptor, PR: progesterone receptor, HER2: human epidermal growth factor receptor-2, CDK4/6i: cyclin-dependent kinase 4/6 inhibitors

Variable, n=158	N (%)	Denosumab (57)	Zoledronat (101)
Age			
<60	78 (49.4)	32	46
≥60	80 (50.6)	25	55
Menopausal status			
Pre-menopause	43 (72.8)	17	26
Post-menopause	115 (27.2)	40	75
ECOG status (n=150)			
0	101 (63.9)	42	59
1	43 (27.2)	13	30
2	6 (3.8)	1	5
Histological type			
Invasive ductal carcinoma	72 (45.6)	21	51
Invasive lobular carcinoma	21 (13.3)	11	10
Infiltrating carcinoma	60 (38.0)	24	36
Mixt (lobuler and ductal)	5 (3.1)	1	4
ER status % (n=150)			
<40	9 (6.0)	2	7
40-90	41 (27.3)	15	26
>90	100 (66.7)	36	64
PR status			
Positive	138 (87.3)	49	89
Negative	20 (12.7)	8	12
HER2 overexpression (n=148)			
Score 0	71 (48.0)	26	45
Score 1	42 (28.4)	17	25
Score 2 fish negative	35 (23.6)	12	23
Ki-67 proliferation index (n=113)			
<20	38 (33.6)	14	24
≥20	75 (66.4)	28	47
Grade (n=136)			
I	13 (9.6)	6	7
II	101 (74.3)	38	63
III	22 (16.2)	5	17
Disease status			
Recurrent metastatic	83 (52.5)	30	53
De novo metastatic	75 (47.5)	27	48
Endocrine resistance status			
Sensitive	73 (46.2)	28	45
Resistant	85 (53.8)	29	56
CDK4/6i			
Ribociclib	84 (53.2)	35	49
Palbociclib	74 (46.8)	22	52
Hormone therapy			
Letrozole	80 (50.6)	35	45
Fulvestrant	78 (49.4)	22	56
CDK4/6i treatment line			
First line	79 (50.0)	33	46
Second line	54 (34.2)	17	37
Third and beyond	25 (15.8)	7	18
Isolated bone metastasis			
Presence	64 (40.5)	27	37
Absence	94 (59.5)	30	64
Visceral metastasis status (n=156)			
Presence	64 (41.0)	24	40
Absence	92 (59.0)	33	59
Presence of progression			
Presence	97 (61.4)	32	65
Absence	61 (38.6)	25	36
Latest status			
Exitus	60 (62.0)	16	44
Alive	98 (38.0)	41	57

**Figure 1:**
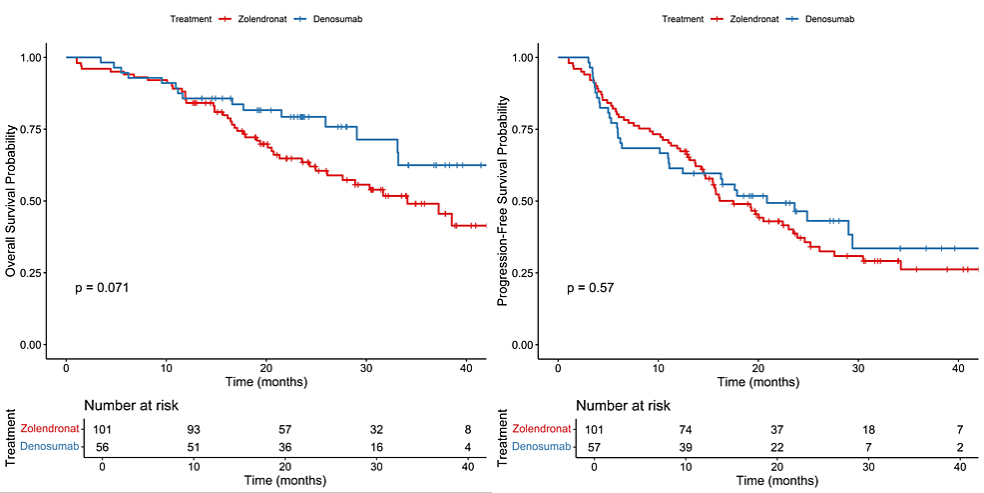
Kaplan-Meier survival curves for denosumab and ZA ZA: zoledronic acid

**Figure 2:**
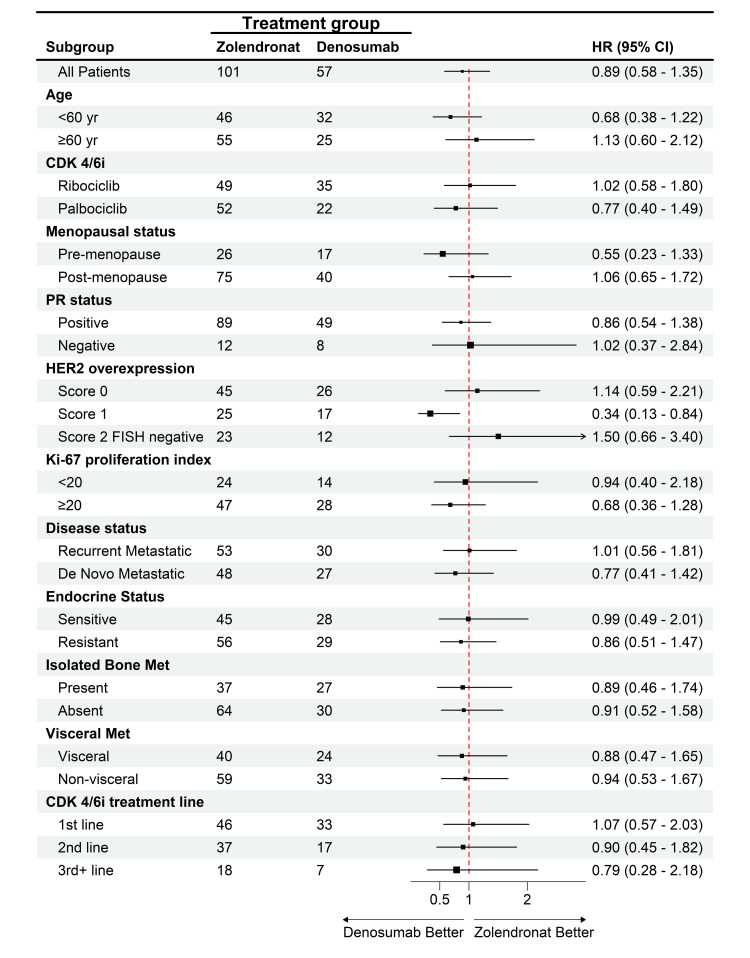
HR analysis for PFS across subgroups in denosumab vs. ZA therapy HR: hazard ratio, PFS: progression-free survival, ZA: zoledronic acid, CDK4/6i: cyclin-dependent kinase 4/6 inhibitors, PR: progesterone receptor, HER2: human epidermal growth factor receptor-2

The median PFS was 20.8 months among patients in the denosumab group, as compared with 17.5 months among patients in the ZA group (HR: 0.89; 95% CI, 0.58-1.35, p=0.57). Although denosumab had better PFS and OS than ZA, the Kaplan-Meier curve showed no significant differences between the two drugs (Figure 1). The HR didn’t differ significantly across subgroups defined according to stratification factors and other baseline characteristics except for patients with a score of 1 HER2 overexpression. Patients taking denosumab had significantly improved PFS compared to ZA (HR: 0.34, %95-CI: 0.13-0.84, p=0.01) (Figure 2). While the mPFS of patients receiving denosumab with a HER2 score of 1 was 21.1 months, the mPFS of patients receiving ZA was 16 months.

**Figure 3:**
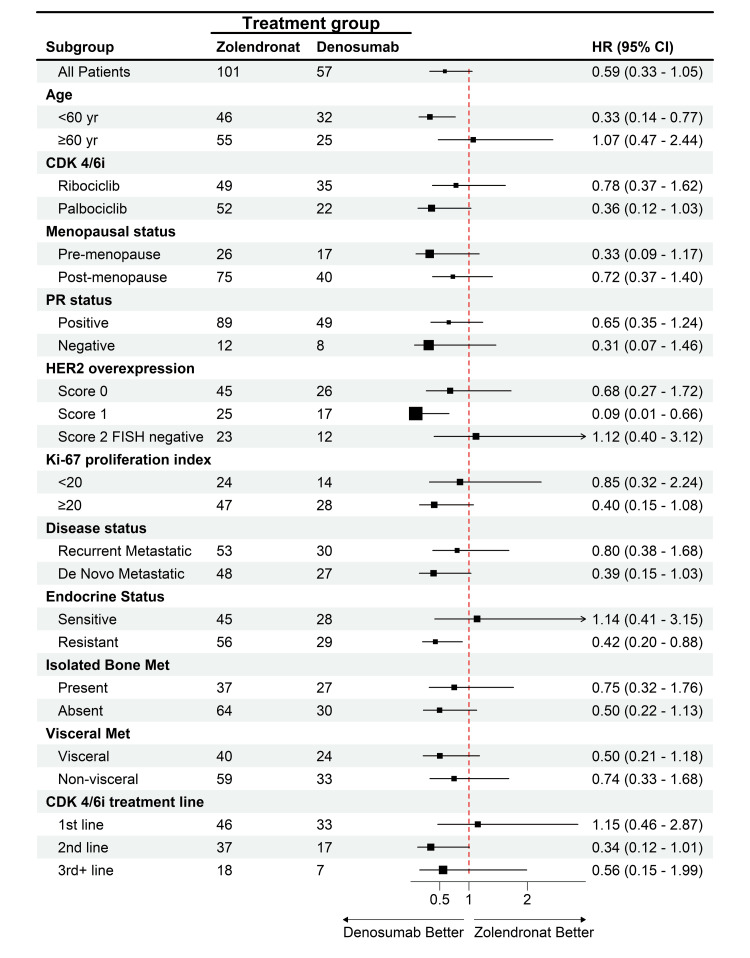
HR analysis for OS across subgroups in denosumab vs. ZA therapy HR: hazard ratio, OS: overall survival, ZA: zoledronic acid, CDK4/6i: cyclin-dependent kinase 4/6 inhibitors, PR: progesterone receptor, HER2: human epidermal growth factor receptor-2

Meanwhile, mOS was not achievable in patients in the denosumab group, while it was 34.1 months in patients in the ZA group (HR: 0.59; 95% CI, 0.33 to 1.05, p=0.07). The HR showed a significant improvement for the denosumab group in patients under 60 of age (HR: 0.33, %95-CI: 0.14-0.77, p<0.01), patients with a score of 1 HER2 overexpression (HR: 0.09, %95-CI: 0.01-0.66, p=0.01), and patients with resistant endocrine (HR: 0.42, %95-CI: 0.20-0.88, p=0.02) compared to the ZA group with no significant differences in the other subgroups (Figure 3).

**Figure 4:**
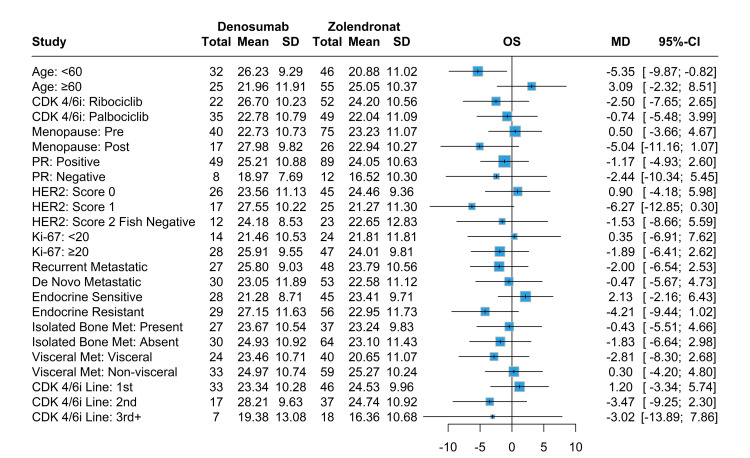
Mean survival analysis for OS in denosumab and ZA treatment groups stratified various baseline characteristics OS: overall survival, ZA: zoledronic acid, CDK4/6i: cyclin-dependent kinase 4/6 inhibitors, PR: progesterone receptor, HER2: human epidermal growth factor receptor-2

**Figure 5:**
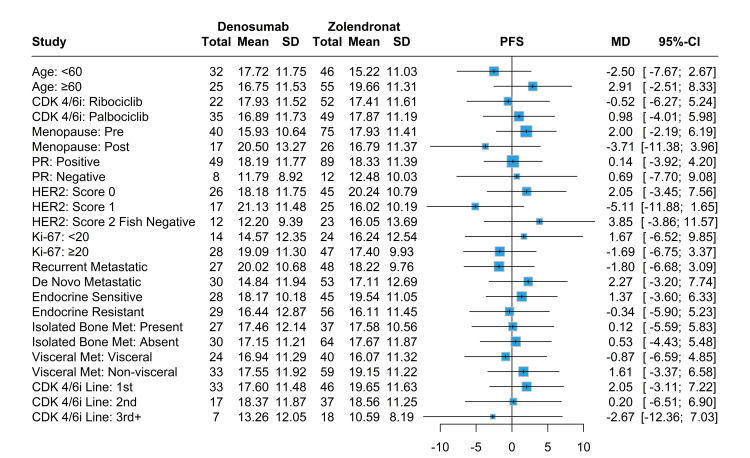
Mean survival analysis for PFS in denosumab and ZA treatment groups stratified various baseline characteristics PFS: progression-free survival, ZA: zoledronic acid, CDK4/6i: cyclin-dependent kinase 4/6 inhibitors, PR: progesterone receptor, HER2: human epidermal growth factor receptor-2

**Figure 6:**
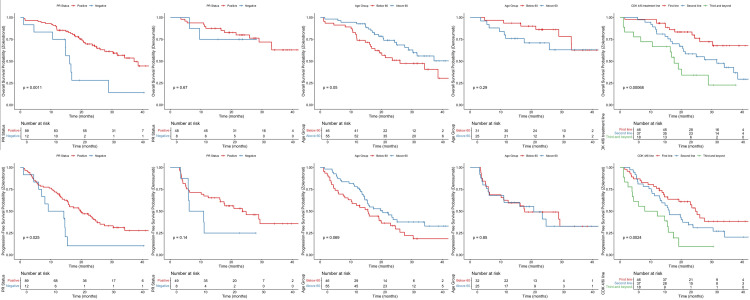
Mean survival analysis for PFS and OS in denosumab and ZA treatment groups stratified various baseline characteristics (PR status, age, CDK4/6i treatment line) PFS: progression-free survival, OS: overall survival, PR: progesterone receptor, ZA: zoledronic acid, CDK4/6i: cyclin-dependent kinase 4/6 inhibitors

**Figure 7:**
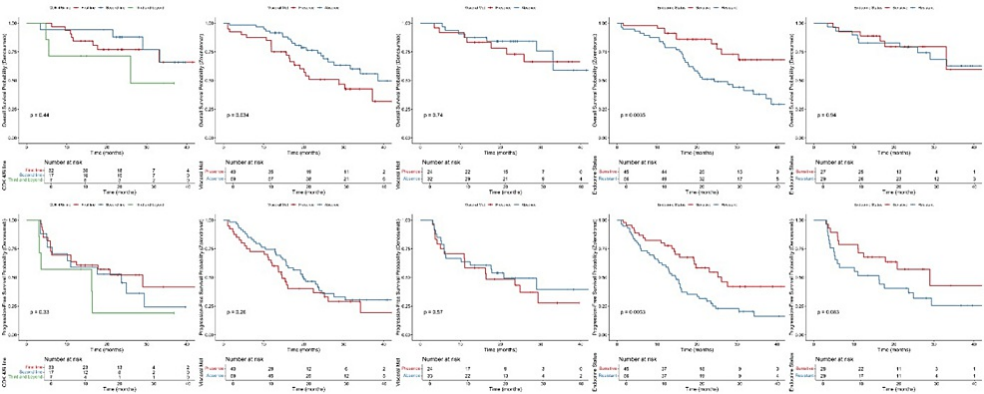
Mean survival analysis for PFS and OS in denosumab and ZA treatment groups stratified various baseline characteristics (CDK4/6i treatment line, visceral metastasis, endocrine resistance status) PFS: progression-free survival, OS: overall survival, ZA: zoledronic acid, CDK4/6i: cyclin-dependent kinase 4/6 inhibitors

In terms of mean survival for both PFS and OS between denosumab and ZA, only patients under age 60 had better OS, with patients taking denosumab having a mean survival of 26.2 months compared to 20.9 months in patients taking ZA (Figures 4-5). Patients who had positive PR status and first-line CDK4/6i and were endocrine-sensitive had better PFS while taking ZA. Similarly, patients who had positive PR status and first-line CDK4/6i, the absence of visceral metastases, and were endocrine-sensitive had better OS while taking ZA. The denosumab group had significant improvement in both PFS and OS for score 1 HER2 overexpression compared to score 0 and score 2 FISH negative, with no significant differences between other variables nor any in terms of denosumab use (Figures 6-7).

**Figure 8:**
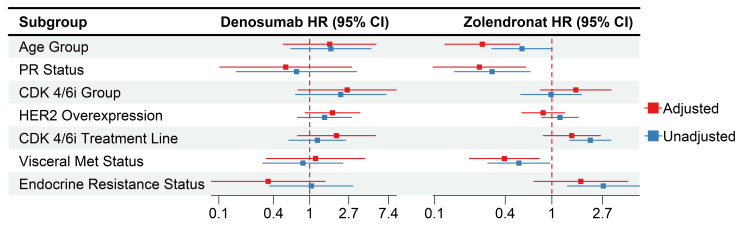
Adjusted HR for the denosumab group across various baseline characteristics (OS) HR: hazard ratio, OS: overall survival, PR: progesterone receptor, HER2: human epidermal growth factor receptor-2, CDK4/6i: cyclin-dependent kinase 4/6 inhibitors

**Figure 9:**
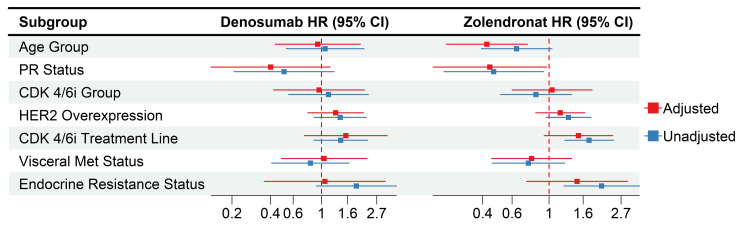
Adjusted HR for the ZA group across various baseline characteristics (PFS) HR: hazard ratio, ZA: zoledronic acid, PFS: progression-free survival, PR: progesterone receptor, HER2: human epidermal growth factor receptor-2, CDK4/6i: cyclin-dependent kinase 4/6 inhibitors

After adjustment, the ZA group showed a significantly better OS for age group over 60 (HR: 0.26, 95% CI: 0.12-0.53), also still had significantly better OS for positive PR status (HR: 0.24, %95 CI: 0.10-0.60) and absence of visceral metastases (HR: 0.39, 95% CI: 0.20-0.78), while the first-line CDK4/6i and endocrine-sensitive groups' significant HRs had diminished. As for denosumab, the adjusted HRs were similar to the unadjusted ones, with no significant differences (Figure 8). The ZA-adjusted HRs showed significantly better PFS for the patients above the age of 60 (HR: 0.42, 95% CI: 0.24-0.74) and those with positive PR status (HR: 0.44, 95% CI: 0.20-0.96). Furthermore, similar to the OS, the first-line CDK4/6i and endocrine-sensitive groups' significant HRs were diminished following adjustment for other variables. Additionally, there were no significant differences within the denosumab group (Figure 9).

## Discussion

Although there is a substantial body of pre-clinical evidence indicating that RANK signaling promotes the proliferation and metastatic progression of BC [4,18,19], there is still considerable debate as to whether the targeted inhibition of RANK signaling by denosumab treatment will confer clinical benefits in patients with early BC. While the ABCSG-18 trial demonstrated that the addition of denosumab to adjuvant systemic treatment resulted in improved disease-free survival [20], the D-CARE trial did not identify any improvement in disease-related outcomes for high-risk early BC patients treated with denosumab [21].

Furthermore, the current study reported that RANK overexpression drives intrinsic resistance to CDK4/6i, which is associated with a decreased proliferation rate and an aberrant interferon response in tumor cells. The study focused on the therapeutic potential of RANK pathway inhibition through RANKL targeting, demonstrating that it not only sensitizes RANK overexpression luminal BC cells to CDK4/6i in vitro and in vivo. Furthermore, it has been demonstrated that this approach effectively restores the sensitivity of cells that have acquired resistance and prevents its onset when used in combination with CDK4/6i [22]. The results of the survival analysis in our study indicated a significant association between denosumab treatment and a more favorable OS and PFS in comparison to ZA treatment for BM. Three studies with similar designs have directly compared denosumab with ZA in patients with BC [23], prostate cancer [24], and BM related to other solid tumors [25]. A meta-analysis of these three phase III trials concluded that OS and disease progression rates were similar for both treatments [26]. Nevertheless, other researchers have reported that denosumab leads to better OS compared to ZA [27]. These findings are in accordance with the survival data previously reported from a trial comparing the efficacy and safety of denosumab and ZA in patients with lung cancer [27]. In fact, a PFS contribution of approximately five months was observed in patients with a HER2 score of 1, and an OS contribution of approximately five months was observed in patients under the age of 60. Besides, the HR showed a significant improvement for the denosumab group in patients with resistant endocrine (HR: 0.42, %95-CI: 0.20-0.88, p=0.02) compared to the ZA group. While mOS could not be achieved in patients receiving denosumab with endocrine resistance, mOS was 24.3 months in patients receiving ZA. Based on this result, we think that denosumab shows its effectiveness against endocrine resistance.

Since there were no previous similar studies, in the subgroup analysis of patients receiving antiresorptive therapy, being over 60 years of age (25 months vs. 20 months, respectively) and PR positive (24 months vs. 16 months, respectively) among those receiving zoledronat resulted in statistically significant OS. Again, in terms of PFS, being over 60 years of age (19 months vs. 15 months, respectively) and being PR positive (18 months vs. 12 months, respectively) created a statistically significant difference in PFS. In patients receiving denosumab, HR was similar after adjusted analysis, and there was no subgroup difference in OS and PFS. Therefore, when choosing antiresorptive treatments in terms of patients' creatinine clearance and quarterly applicability, it can be considered that zoledronat OS and PFS contribution will be as much as denosumab in patients over 60 years of age and PR positive. On the other hand, denosumab should be prioritized in all other subgroups.

The limitations of this study are as follows: firstly, the study was retrospective and non-randomized in nature and was conducted at a single institution. Secondly, the sample size was relatively small. Finally, the expression of potential biomarkers, such as RANKL and RANK, in tumor cells was not investigated.

## Conclusions

Considering that RANK pathway status may be a predictive biomarker for CDK4/6i treatment and may cause treatment resistance, our results demonstrate the clinical relevance of the combination of CDK4/6i + ET with RANKL inhibition. This study highlights the potential clinical relevance of the RANK pathway in aBC treatment, and our findings suggest that denosumab may offer significant benefits in terms of PFS and OS for certain subgroups, particularly those with HER2 scores of 1, patients under 60, and those with endocrine-resistant aBC. These results highlight the potential of RANKL inhibition to enhance the therapeutic efficacy of CDK4/6 inhibitors, offering a promising avenue for overcoming resistance and improving clinical outcomes in aBC. Further prospective, randomized studies with larger sample sizes are needed to validate these findings and explore the mechanistic underpinnings of RANK pathway involvement in BC progression and treatment resistance.
